# Medical therapy following hospitalization for heart failure with reduced ejection fraction and association with discharge to long-term care: a cross-sectional analysis of the REasons for Geographic And Racial Differences in Stroke (REGARDS) population

**DOI:** 10.1186/s12872-017-0682-3

**Published:** 2017-09-16

**Authors:** Emily B. Levitan, Melissa K. Van Dyke, Ligong Chen, Raegan W. Durant, Todd M. Brown, J. David Rhodes, Olusola Olubowale, Oluwole Muyiwa Adegbala, Meredith L. Kilgore, Justin Blackburn, Karen C. Albright, Monika M. Safford

**Affiliations:** 1 University of Alabama at Birmingham, Birmingham, AL USA; 2 Amgen Inc, Thousand Oaks, CA USA; 3 Weill Cornell Medicine, New York, NY USA; 4 Department of Epidemiology, University of Alabama at Birmingham, 1720 2nd Ave S, RPHB 220, Birmingham, AL 35294-0022 USA

**Keywords:** Heart failure, Therapy, Long-term care

## Abstract

**Background:**

Less intensive treatment for heart failure with reduced ejection fraction (HFrEF) may be appropriate for patients in long-term care settings because of limited life expectancy, frailty, comorbidities, and emphasis on quality of life.

**Methods:**

We compared treatment patterns between REasons for Geographic And Racial Differences in Stroke (REGARDS) study participants discharged to long-term care versus home following HFrEF hospitalizations. We examined medical records and Medicare pharmacy claims for 147 HFrEF hospitalizations among 80 participants to obtain information about discharge disposition and medication prescriptions and fills.

**Results:**

Discharge to long-term care followed 22 of 147 HFrEF hospitalizations (15%). Participants discharged to long-term care were more likely to be prescribed beta-blockers and less likely to be prescribed aldosterone receptor antagonists and hydralazine/isosorbide dinitrate (96%, 14%, and 5%, respectively) compared to participants discharged home (81%, 22%, and 23%, respectively). The percentages of participants discharged to long-term care and home who had claims for filled prescriptions were similar for beta-blockers (68% versus 66%) and angiotensin converting enzyme inhibitors or angiotensin receptor blockers (ACEI/ARBs) (45% versus 47%) after 1 year. Smaller percentages of participants discharged to long-term care had claims for filled prescriptions of other medications compared to participants discharged home (diuretics: long-term care-50%, home-72%; hydralazine/isosorbide dinitrate: long-term care-5%, home-23%; aldosterone receptor antagonists: long-term care-5%, home-23%).

**Conclusions:**

Differences in medication prescriptions and fills among individuals with HFrEF discharged to long-term care versus home may reflect prioritization of some medical therapies over others for patients in long-term care.

## Background

Heart failure (HF) is common in populations residing in long-term care settings, with estimates of HF prevalence ranging from a fifth to a third of all patients in long-term care facilities [1–6]. In the Get With The Guidelines-HF registry, one in five hospitalized HF patients were discharged to skilled nursing facilities, and these patients had higher rates of mortality and rehospitalization than patients discharged home [7]. Guideline-concordant pharmacologic therapy is generally recommended for patients with HF discharged to long-term care [4]. For patients with HF with reduced ejection fraction (HFrEF), diuretics—particularly loop diuretics, angiotensin converting enzyme inhibitors or angiotensin receptor blockers (ACEI/ARB), beta-blockers—particularly carvedilol, metoprolol succinate, and bisoprolol (HFrEF recommended beta-blockers), and aldosterone receptor antagonists may improve quality of life and reduce mortality and rehospitalization [8]. Hydralazine and isosorbide dinitrate in combination are recommended for black individuals already receiving maximal beta-blocker and ACEI therapy or for persons of any race unable to tolerate an ACEI or an ARB [8]. With the exception of diuretics, the effectiveness of recommended pharmacologic therapies is supported by large, high-quality randomized controlled clinical trials [8]. However, none of the trials included patients in long-term care, and these patients may be more susceptible to side effects and have limited life expectancy [4]. These characteristics suggest that less aggressive treatment for HFrEF could be appropriate for many patients discharged to long-term care. In particular, for individuals with limited life expectancy, therapies which improve quality of life and reduce hospitalization may be prioritized above therapies that prolong life at the expense of greater side effects. A recent statement from the American Heart Association and the Heart Failure Society of America emphasized individualized care planning for patients with HF in long-term care, but differences in treatment for those individuals with HF discharged to long-term care versus discharged to home are not well-described [4]. In this study, we examined medication treatment patterns among black and white participants of the REasons for Geographic And Racial Differences in Stroke (REGARDS) study who experienced HFrEF hospitalizations and compared treatment patterns between participants discharged to long-term care and those discharged home.

## Methods

### Study population

The REGARDS study population includes 30,239 white and black women and men 45 years of age and older at the time of study entry (2003–2007) from across the 48 continental United States (US) [9]. Potentially eligible individuals were identified through commercial lists. The study was designed to investigate the racial and regional differences in stroke mortality; black individuals and residents of the US Stroke Belt (North Carolina, South Carolina, Georgia, Alabama, Arkansas, Louisiana, Mississippi, and Tennessee) were oversampled. Participants completed a telephone interview followed by a home visit conducted by a trained health professional. We linked participants’ information to Medicare claims using social security number, date of birth, and sex [10].

For this study, we included REGARDS participants who had one or more Medicare claims for hospitalizations with HF as the primary discharge diagnosis (International Classification of Diseases, 9th edition, (ICD-9) diagnosis codes 402.01, 402.11, 402.91, 404.01, 404.03, 404.11, 404.13, 404.91, 404.93, 428.0, 428.1, 428.20, 428.21, 428.22, 428.23, 428.30, 428.31, 428.32, 428.33, 428.40, 428.41, 428.42, 428.43, or 428.9) in 2006–2011 and Medicare inpatient and prescription drug coverage at the time of hospitalization. Multiple hospitalizations could be included for each individual. We identified 612 HF hospitalization claims, from which records were retrieved and abstracted for 400 hospitalizations among 232 unique participants. Study investigators (OO, OMA, JDR) abstracted information on comorbidities, ejection fraction, in-hospital treatment, and discharge disposition using a standardized data abstraction form. HFrEF hospitalizations were defined as those with documented ejection fraction <40%, a qualitative report of low ejection fraction, or a Medicare ICD-9 diagnosis specifying systolic dysfunction (428.2x or 428.4x) during the current hospitalization or, in the absence of information about ejection fraction during the current hospitalization, a history of ejection fraction <40% or qualitative report of low ejection fraction during a prior medical encounter. We excluded 56 hospitalizations without discharge medication lists in the retrieved medical records, 152 hospitalizations for HF with preserved ejection fraction and 45 hospitalizations without documentation of ejection fraction. The analytic dataset included 147 hospitalizations among 80 unique individuals with HFrEF where the participant was discharged alive and hospitalization records with discharge medication lists could be retrieved.

This research was supported by an academic collaboration between University of Alabama at Birmingham and Amgen, Inc. The funders provided comments on the design and interpretation of this work. The academic authors conducted all analyses and maintained the rights to publish this manuscript.

### Discharge disposition

Discharge disposition was determined based on review of medical records. Discharge disposition was categorized as discharged to home, long-term care, or hospice; no participants were discharged to hospice. Long-term care included skilled nursing facilities/nursing homes and rehabilitation facilities.

### Covariates

Information on age, sex, and race was collected during the REGARDS baseline interview [9]. History of asthma, chronic obstructive pulmonary disease (COPD), atrial fibrillation or flutter, peripheral vascular disease, stroke, hypertension, hypotension, diabetes, chronic kidney disease, dialysis, implanted cardiac devices, myocardial infarction, coronary heart disease, coronary heart disease events in the prior year, prior heart failure diagnosis, blood pressure at discharge, and ejection fraction were determined by medical record review. Conditions were considered to be present if there was documentation of the diagnosis in medical records (e.g., notation of hypotension). Additionally, medical record reviewers recorded whether suspected COPD exacerbation contributed to the current hospitalization and whether participants experienced hypotension during the current hospitalization. Hospitalization in the 30 days and 1 year prior to current admission, presence of outpatient clinic visits in the week after discharge, and mortality in the year following discharge were assessed using Medicare claims and enrollment information. Nursing home residence was defined as Medicare claims for care delivered in a nursing facility in the prior year; these claims are distinct from claims for the Medicare skilled nursing facility benefits that are available to beneficiaries following a hospitalization [11].

### Medication prescriptions and fills

Information on prescribed medications including dose was abstracted from admission and discharge medication lists. We conducted follow-up for medication prescription fills through Medicare Part D prescription drug claims, which include information on dose, for up to one year following HFrEF hospitalization. Medicare covers up to 100 days of skilled nursing care, including prescription medications, following a hospitalization [12]. During the study period, Part D claims were submitted inconsistently during post-hospitalization periods of Medicare-covered skilled nursing care [13]. Therefore, we did not examine short-term differences in medication filling patterns.

The medications of interest included all beta-blockers, specific beta-blockers recommended for HFrEF (carvedilol, bisoprolol, or metoprolol succinate), ACEI/ARBs, diuretics, loop diuretics, and aldosterone receptor antagonists because these medications are recommended for most patients with HFrEF [8]. Because more than 40% of REGARDS participants are black [9], we additionally examined use of hydralazine in combination with isosorbide dinitrate.

### Statistical analysis

We calculated means and standard deviations or numbers and percentages of demographic characteristics, comorbidities, and hospitalization characteristics overall and stratified by whether the participant was discharged to long-term care. For each medication category, we calculated the proportion with the medication on the admission and discharge medication lists and the proportion with Medicare claims for the medications within 1 year. Additionally, we calculated the percentage of the guideline-recommended target dose of specific beta-blockers recommended in HFrEF (carvedilol, bisoprolol, or metoprolol succinate) and ACEI/ARBs prescribed at discharge and filled within 1 year. These values were calculated as the dose prescribed or filled of each specific agent divided by the target dose for that agent recommended in the 2013 American College of Cardiology/American Heart Association guideline for the management of HF [8].

For each of the medications, we used generalized estimating equation Poisson models with robust variance estimators to calculate risk ratios for presence of the medications on the admission and discharge medication lists and 1-year prescription fills associated with discharge to long-term care. The generalized estimating equation approach accounted for correlations between multiple hospitalizations for a single individual. Models were adjusted for age, race, and sex.

To account for missing data in some covariates (range 0–28% missingness), we used fully conditional specification methods to impute 20 complete datasets [14]. Analyses were conducted in each imputed dataset and point estimates and standard errors were combined across datasets accounting for the uncertainty in the imputed data [15]. All analyses were conducted using SAS version 9.3 (Cary, NC).

## Results

REGARDS participants were discharged to long-term care following 22 of 147 HFrEF hospitalizations (15%). Black participants were less likely to be discharged to long-term care than white participants (9% versus 23%). Participants discharged to long-term care were older, more likely to be residents of nursing homes prior to the HFrEF admission, and more likely to have been hospitalized in the 30 days and 1 year prior to the HFrEF admission (Table 1).

**Table 1:** Characteristics of HFrEF hospitalizations among participants in the REasons for Geographic and Racial Differences in Stroke (REGARDS) study by discharge status

	Overall (*n* = 147)	Discharged to long-term care (*n* = 22)	Not discharged to long-term care (*n* = 125)
Demographics			
Age			
< 65 years	26 (18%)	1 (5%)	25 (20%)
65–69 years	28 (19%)	2 (9%)	26 (21%)
70–74 years	30 (20%)	0	30 (24%)
75–79 years	22 (15%)	4 (18%)	18 (14%)
80–84 years	21 (14%)	9 (41%)	12 (10%)
≥ 85	20 (14%)	6 (27%)	14 (11%)
Sex			
Female	61 (41%)	9 (41%)	52 (42%)
Male	86 (59%)	13 (59%)	73 (58%)
Race			
White	61 (41%)	14 (64%)	47 (38%)
Black	86 (59%)	8 (36%)	78 (62%)
Nursing home resident	14 (10%)	7 (32%)	7 (6%)
Comorbidities			
Asthma	20 (14%)	3 (14%)	17 (14%)
Chronic obstructive pulmonary disease	36 (24%)	6 (27%)	30 (24%)
Atrial fibrillation/ atrial flutter	63 (43%)	12 (55%)	51 (41%)
Peripheral vascular disease	34 (23%)	4 (18%)	30 (24%)
Stroke	29 (20%)	4 (18%)	25 (20%)
Hypertension	140 (95%)	20 (91%)	120 (96%)
Hypotension history	12 (8%)	3 (14%)	9 (7%)
Diabetes	96 (65%)	10 (45%)	86 (69%)
Chronic kidney disease	69 (47%)	11 (50%)	58 (46%)
Dialysis	22 (15%)	4 (18%)	18 (14%)
Implanted cardiac device	54 (37%)	7 (32%)	47 (38%)
Myocardial infarction	53 (36%)	8 (36%)	45 (36%)
Coronary heart disease history	113 (77%)	18 (82%)	95 (76%)
Coronary heart disease event within past 12 months	15 (10%)	2 (9%)	13 (10%)
Prior heart failure diagnosis	137 (93%)	21 (95%)	116 (93%)
Hospitalization in 30 days before admission	34 (23%)	9 (41%)	25 (20%)
Hospitalization in 1 year before admission	112 (76%)	18 (82%)	94 (75%)
Hospitalization information			
Ejection fraction, mean ± standard deviation (%)	33 ± 29	31 ± 32	33 ± 29
Current hypotension	28 (19%)	4 (18%)	24 (19%)
Systolic blood pressure at discharge mean ± standard deviation (mmHg)	124 (24)	121 (28)	125 (24)
Diastolic blood pressure at discharge mean ± standard deviation (mmHg)	71 (15)	69 (17)	71 (14)
Chronic obstructive pulmonary disease exacerbation contributed to admission	11 (7%)	3 (14%)	8 (6%)
Outpatient clinic visit in the week after discharge	60 (41%)	14 (64%)	46 (37%)
Mortality within 1 year of discharge	49 (33%)	9 (41%)	40 (32%)

**Fig. 1:**
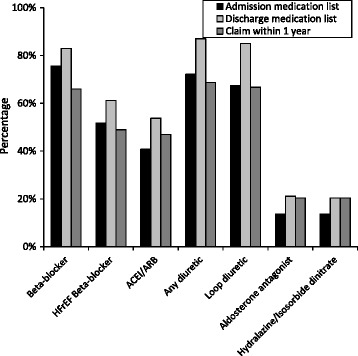
Medication use among REasons for Geographic And Racial Differences in Stroke study participants hospitalized for heart failure with reduced ejection fraction. HFrEF: Heart failure with reduced ejection fraction; ACEI/ARB: Angiotensin converting enzyme inhibitor or angiotensin receptor blocker. Pre-admission and discharge use were determined using medical record review. Claims within 1 year were assessed using Medicare data

Pre-admission use of medications, obtained from the admission medication lists, varied from 76% using beta-blockers to 14% using aldosterone antagonists and hydralazine in combination with isosorbide dinitrate (Fig. 1). When stratified by race, 17% of black participants and 7% of white participants used hydralazine in combination with isosorbide dinitrate. All of the medications were more common on discharge than admission medication lists with percentages ranging from 87% recommended diuretics to 20% hydralazine in combination with isosorbide dinitrate (28% of black participants and 10% of white participants) at discharge. For beta-blockers, ACEI/ARBs, and diuretics, the percentages of participants with evidence of prescriptions filled after follow-up for up to 1 year was substantially lower than the percentages with the medications included on the discharge medication lists.

Participants discharged to long-term care were more likely to have used beta-blockers and diuretics and less likely to have used hydralazine in combination with isosorbide dinitrate and aldosterone receptor antagonists prior to the HFrEF hospitalization compared to those discharged home (Table 2). At discharge, participants discharged to long-term care were more likely to have been prescribed beta-blockers and less likely to have been prescribed hydralazine in combination with isosorbide dinitrate and aldosterone receptor antagonists. For beta-blockers and ACEI/ARBs, the percentages of participants discharged to long-term care and home who had claims for filled prescriptions were similar after 1 year. Smaller percentages of participants discharged to long-term care had claims for filled prescriptions of other medications after 1 year compared to participants discharged home. After adjusting for age, race, and sex, participants discharged to long-term care were more likely to have beta-blockers and less likely to have aldosterone receptor antagonists and hydralazine in combination with isosorbide dinitrate on their discharge medication lists than those discharged home, although associations were not statistically significant (Table 3). For beta-blockers and ACEI/ARBs, the groups had similar proportions of fills at 1 year. Claims for diuretics, aldosterone receptor antagonists, and hydralazine in combination with isosorbide dinitrate fills remained lower among participants discharged to long-term care at 1 year. Among those prescribed and filling the medications, the average doses of HFrEF-recommended beta-blockers and ACEI/ARBs were lower among participants discharged to long-term care than those discharged home (Table 4). The average percentage of the guideline-recommended target doses of beta-blockers and ACEI/ARBs filled at 1 year was lower than the average percentage of the target dose indicated on the discharge prescription lists.

**Table 2:** Medication use among REGARDS participants hospitalized for HFrEF

	Admission medication list^a^	Discharge medication list^a^	Claims within 1 year^b^
Beta-blocker			
Discharged home	74%	81%	66%
Discharged to long-term care	82%	95%	68%
HFrEF beta-blocker^c^			
Discharged home	52%	60%	49%
Discharged to long-term care	50%	68%	50%
ACEI/ARB			
Discharged home	41%	54%	47%
Discharged to long-term care	41%	55%	45%
Diuretic			
Discharged home	71%	87%	72%
Discharged to long-term care	77%	86%	50%
Loop diuretic			
Discharged home	66%	85%	70%
Discharged to long-term care	77%	86%	50%
Hydralazine in combination with isosorbide dinitrate			
Discharged home	14%	23%	23%
Discharged to long-term care	9%	5%	5%
Aldosterone receptor antagonist			
Discharged home	14%	22%	23%
Discharged to long-term care	9%	14%	5%

*HFrEF* Heart failure with reduced ejection fraction, *ACEI/ARB* Angiotensin converting enzyme inhibitor or angiotensin receptor blocker

^a^Determined from review of medical records

^b^Determined using Medicare pharmacy claims data

^c^Carvedilol, metoprolol succinate, or bisoprolol

**Table 3:** Age, race, and sex adjusted risk ratios for the associations between discharge to long-term care and medication use among REGARDS participants hospitalized for HFrEF

	Admission medication list^a^	Discharge medication list^a^	Claims within 1 year^b^
Beta-blocker	1.1 (0.9, 1.4)	1.2 (1.0, 1.4)	1.0 (0.7, 1.4)
HFrEF beta-blocker^c^	1.0 (0.6, 1.7)	1.3 (0.9, 1.9)	1.0 (0.6, 1.8)
ACEI/ARB	1.0 (0.6, 1.6)	1.0 (0.6, 1.6)	1.0 (0.6, 1.7)
Any diuretic	1.1 (0.8, 1.4)	1.0 (0.8, 1.2)	0.7 (0.4, 1.1)
Loop diuretic	1.2 (0.9, 1.6)	1.0 (0.8, 1.2)	0.7 (0.4, 1.1)
Aldosterone receptor antagonist	0.6 (0.1, 2.3)	0.7 (0.2, 2.2)	0.2 (0.0, 1.4)
Hydralazine in combination with isosorbide dinitrate	0.9 (0.2, 3.7)	0.2 (0.0, 1.3)	0.2 (0.0, 1.8)

*HFrEF* Heart failure with reduced ejection fraction, *ACEI/ARB* Angiotensin converting enzyme inhibitor or angiotensin receptor blocker

^a^Determined from review of medical records

^b^Determined using Medicare claims data

^c^Carvedilol, metoprolol succinate, or bisoprolol

**Table 4:** Average percentage of target dose of medications^a^ among REGARDS participants hospitalized for HFrEF

	Discharge medication list^b^	Claims within 1 year^c^
HFrEF beta-blocker^d^		
Discharged home	35%	23%
Discharged to long-term care	22%	20%
ACEI/ARB		
Discharged home	153%	86%
Discharged to long-term care	63%	50%

*HFrEF* Heart failure with reduced ejection fraction, *ACEI/ARB* Angiotensin converting enzyme inhibitor or angiotensin receptor blocker

^a^Calculated as the dose prescribed or filled of the specific agent divided by the target dose for that agent recommended in the 2013 American College of Cardiology/American Heart Association guideline for the management of heart failure

^b^Determined from review of medical records among those with the medication on their discharge medication

^c^Determined using Medicare pharmacy claims data among those with claims for the medication

^d^Carvedilol, metoprolol succinate, or bisoprolol

## Discussion

In this population of black and white US adults hospitalized for HFrEF, we found that the majority were prescribed beta-blockers and diuretics at discharge, regardless of discharge disposition. Many individuals received beta-blockers other than the 3 that have been shown to have benefit for patients with HFrEF in clinical trials. ACEI/ARBs were prescribed for approximately half and aldosterone receptor antagonists and hydralazine in combination with isosorbide dinitrate were prescribed for approximately one fifth of participants, also with little difference between participants discharged to long-term care and those discharged home. The proportion of participants with Medicare claims for beta-blockers and ACEI/ARBs was similar across discharge status, but the proportion with claims for other medications was smaller among participants discharged to long-term care. The proportion of REGARDS participants with HFrEF discharged to long-term care was lower than the proportion discharged to long-term care in Get With The Guidelines-HF registry [7]. More than half of the HFrEF hospitalizations occurred among black REGARDS participants who were less likely than white participants to be discharged to long-term care, consistent with other US studies [16].

Pharmacologic therapies for HFrEF can impose a substantial burden of side effects, costs, and need for medical care. In a recent analysis, 82% of patients discharged following a hospitalization for HFrEF were eligible to initiate at least 1 new medication and 32% were eligible to initiate 3 or more medications [17]. Patients with HFrEF, particularly those in long-term care, often have multiple comorbidities that may also warrant pharmacologic therapy [7, 18]. In clinical practice, patients with HFrEF are often older, frailer, and have more comorbidities than participants in the landmark clinical trials of HFrEF therapy [19, 20]. Age, frailty, and comorbidities such as chronic kidney disease and COPD may decrease patients’ ability to tolerate medications, alter the goals and priorities of care compared to clinical trial populations, and the applicability or perceived applicability of guidelines [4, 21]. The same patient characteristics that can limit tolerability of medications and change priorities of care also increase the probability that patients are discharged to long-term care following a HFrEF hospitalization [4, 7]. Patients discharged to long-term care following hospitalization are a mix of patients who are expected to return to independent living following a limited period of skilled nursing care, patients who will continue to require assistance with activities of daily living, and those whose ability to live independently is uncertain [4]. We were not able to distinguish between those subgroups in this study, though one third of participants discharged to long term care had been nursing home residents in the prior year. Management of HFrEF in residents of long-term care facilities requires balancing the potential cardiac benefits of therapy with risk of side effects and treatment burden, particularly among patients nearing the end of life [4]. The close observation available in a skilled nursing facility could allow providers to up-titrate medications, especially if the patient anticipates returning to independent living. However, aggressive treatment of HFrEF may not be a priority for patients in long-term care and their providers. In this study we found little difference in the medications prescribed for participants discharged to long-term care and home following a hospitalization for HFrEF, though doses of HFrEF-recommended beta-blockers and ACEI/ARBs were somewhat lower among those discharge to long-term care and Medicare claims for fills of diuretics, aldosterone receptor antagonists, and hydralazine in combination with isosorbide dinitrate were less common among participants discharged to long-term care.

Differences between the REGARDS population with hospitalizations occurring between 2006 and 2011 and previously studied populations likely reflect secular trends of increasing acceptance and use of these medications among patients with HFrEF both for individuals discharged to long-term care and home [17, 22–30]. In a previous study from the Get With The Guideline-HF registry including hospitalizations that occurred in 2005–2006, the percentages of eligible patients prescribed ACEI/ARBs (30.5%) and beta-blockers (33.2%) were lower than in the current study, and patients discharged to skilled nursing facilities were less likely to have been prescribed these medications [7]. Alabama patients hospitalized for HF in 1994 who were eligible for ACEIs were less likely to receive them if they were nursing home residents [31]. In an evaluation of patients with HF with both preserved and reduced ejection fraction in long-term care between 1992 and 1996, percentages using diuretics, ACEI, and beta-blockers were 45%, 26%, and 4%, respectively [32]. In this study we focused on treatment for HFrEF because the recommended therapies for HFpEF are primarily directed at managing comorbidities and fluid overload [8].

Strengths of this study include the population of black and white adults who were recruited from across the continental US and were hospitalized in a wide range of unselected facilities, information on HFrEF hospitalizations and comorbidities obtained from chart reviews, and the availability of both discharge medication lists, reflecting planned treatments, and pharmacy fills, reflecting the treatments purchased by or on behalf of the participants. However, these results must be interpreted in light of several weaknesses. The study population was small which limited us to primarily descriptive analyses. The small study size precluded extensive multivariable adjustment, increasing the risk of uncontrolled and residual confounding. Additionally, we relied on Medicare claims to identify medication fills following hospitalization. We could not observe pharmacy fills that were not submitted for Medicare payment, particularly during periods when participants were receiving skilled nursing care reimbursed by Medicare immediately following hospitalization. Information on functional status and other factors that could influence discharge disposition was not available. Similarly, we did not have detailed information on some characteristics that could affect pharmacologic therapy. For example, we had information about diagnoses of CKD but not glomerular filtration rate or albuminuria.

## Conclusions

In summary, prescriptions for beta-blockers and diuretics were common, but prescriptions for other recommended medications, including ACEI/ARBs, aldosterone receptor blockers, and hydralazine in combination with isosorbide dinitrate were much less frequent among REGARDS participants hospitalized for HFrEF regardless of discharge disposition. The proportion of participants with Medicare claims for medication fills was substantially lower than the proportion with prescriptions. Fills for beta-blockers and ACEI/ARBs, two medications targeted in performance measures [33], were largely similar in participants with HFrEF discharged to long-term care and those discharged home. Other HFrEF medications were less frequently filled among those discharged to long-term care. The observed differences in medication fills by discharge status could reflect prioritization of therapies for HFrEF patients in long-term care.

## Abbreviations

ACEI: Angiotensin converting enzyme inhibitors
ARB: Angiotensin receptor blockers
HF: Heart failure
HFrEF: Heart failure with reduced ejection fraction
ICD-9: International Classification of Diseases, 9th edition
REGARDS: REasons for Geographic And Racial Differences in Stroke
US: United States
